# 

**Published:** 1844-12

**Authors:** 


*Gossip with our Profession.*—If it be true that "it is now manifest that not only the breathing for any length of time of foul air is productive of many of the most serious, and direct attacks of disease, but that it also preys in innumerable other cases, on the constitution in such a way as prematurely to destroy it:"—that "this has particularly been observed to be the case as regards its effects on the stomach, by diminishing the appetite and weakening the digestive powers to such a degree as ultimately to produce premature old age, and thus shorten the duration of life by many years:"—that "in addition to this, it is notorious that the moral and intellectual faculties are impaired nearly in as great degree as the corporeal functions by the depressing effects of breathing a vitiated atmosphere," how *very* necessary it becomes to have the mouth free from impurities. We take the lines above quoted from a late London review of "Illustrations of the Theory and Practice of Ventilation," by Dr. Reid.

How long will it be before the condition of the mouth will be attended to by *many* (it is now by a *few*) physicians, who have under treatment obscure "nervous" affections? We know a man, now hale, hearty and free from even the appearance of disease, who, a few years ago, came to us with fear and trembling to get our advice upon his teeth, which were extensively decayed—seven of them being too far gone for remedy. Our advice was, to remove these seven and restore such others as required it to artificial soundness. He then said that he doubted his ability to bear the operations—that he was "so nervous" that he would be overcome by the least unusual excitement and "faint away"—sometimes he did so several times a day, &c. &c. He however *did* bear the operations, and never had a "nervous fit" afterwards. His general health (and he was supposed to be consumptive) showed a marked improvement immediately, and continued to improve rapidly until he was well. His natural energy of character (which may be said to have been in abeyance) being restored with his health, he rose rapidly from almost hopelessness to the very enviable station he now occupies as a distinguished member of the bar. . . . Inquiries are often made of us where our dental drill-stock (mentioned some time ago in this Journal) can be procured. Mr. Arnold, cutler, Baltimore, has the skill and authority to make and furnish them to the profession. We fell into the common error of expecting too much from such an instrument; and we would advise those who are not clever manipulators not to procure the one in question; because it is extremely probable they will seldom, if ever, use it. The man whose hands are properly educated, however, may frequently, in a single operation, save as much time with it as would be worth the price of the instrument—some seven or ten dollars. . . . . We think there should be in every Medical College a distinct professorship for teaching students the structure, diseases and treatment of the mouth and teeth. How ridiculously absurd the practice of holding up a tooth before a class of students, some of whom are twenty or thirty feet from the lecturer, and attempting to *show*—and all in a single one-hour lecture—the tooth's structure, shape, position in the mouth, affections, &c. &c.—things, *all* of which, to be understood, must be examined at very short distances, and *some* of which must be examined with a microscope! An evidence of the propriety of our views on this subject, and an example of how little of truth is known and how much of error is taught, even in "high places," upon the affections of the teeth, may be seen in the fact that one of the "lights of science" in one of the most famous medical schools in America, and a most voluminous writer withal, stated, captiously, in a review of a work upon dental surgery, that caries of the teeth *always* commences *within* the substance of the teeth!—a single instance of which no one ever saw. We would not have it supposed from these remarks, that we would have students qualified at medical schools for the practice of dental surgery:—we would have this distinct professorship expressly for medical students: to teach them what it is essential, as well for their credit as for success in the practice of *their* profession, that they should know: and what can be *best,* if not *only,* taught by one who has observed closely and practised long and successfully in Dental Surgery. M.

*Washington, November,* 1844.

